# Therapeutic potential of transplanted placental mesenchymal stem cells in treating Chinese miniature pigs with acute liver failure

**DOI:** 10.1186/1741-7015-10-56

**Published:** 2012-06-06

**Authors:** Hongcui Cao, Jinfeng Yang, Jiong Yu, Qiaoling Pan, Jianzhou Li, Pengcheng Zhou, Yanyuan Li, Xiaoping Pan, Jun Li, Yingjie Wang, Lanjuan Li

**Affiliations:** 1 State Key Laboratory for Diagnosis and Treatment of Infectious Diseases, the First Affiliated Hospital, School of Medicine, Zhejiang University, 79 Qingchun Road, Hangzhou, 310003, P.R. China; 2 Department of Pathology, the First Affiliated Hospital, School of Medicine, Zhejiang University, 79 Qingchun Road, Hangzhou 310003, P.R. China

**Keywords:** Acute liver failure, Cell transplantation, Chinese miniature pig, Irradiation, Placental mesenchymal stem cells, Portal vein

## Abstract

**Background:**

Stem cell-based therapy to treat liver diseases is a focus of current research worldwide. So far, most such studies depend on rodent hepatic failure models. The purpose of this study was to isolate mesenchymal stem cells from human placenta (hPMSCs) and determine their therapeutic potential for treating Chinese experimental miniature pigs with acute liver failure (ALF).

**Methods:**

hPMSCs were isolated and analyzed for their purity and differentiation potential before being employed as the donor cells for transplantation. ALF models of Chinese experimental miniature pigs were established and divided into four groups: no cell transplantation; hPMSCs transplantation via the jugular vein; X-ray-treated hPMSCs transplantation via the portal vein; and hPMSCs transplantation via the portal vein. The restoration of biological functions of the livers receiving transplantation was assessed via a variety of approaches such as mortality rate determination, serum biochemical analysis, and histological, immunohistochemical, and genetic analysis.

**Results:**

hPMSCs expressed high levels of CD29, CD73, CD13, and CD90, had adipogenic, osteogenic, and hepatic differentiation potential. They improved liver functions *in vivo *after transplantation into the D-galactosamine-injured pig livers as evidenced by the fact that ALT, AST, ALP, CHE, TBIL, and TBA concentrations returned to normal levels in recipient ALF pigs. Meanwhile, histological data revealed that transplantation of hPMSCs via the portal vein reduced liver inflammation, decreased hepatic denaturation and necrosis, and promoted liver regeneration. These ameliorations were not found in the other three groups. The result of 7-day survival rates suggested that hPMSCs transplantation via the portal vein was able to significantly prolong the survival of ALF pigs compared with the other three groups. Histochemistry and RT-PCR results confirmed the presence of transplanted human cells in recipient pig livers (Groups III, IV).

**Conclusions:**

Our data revealed that hPMSCs could not only differentiate into hepatocyte-like cells *in vitro *and *in vivo*, but could also prolong the survival time of ALF pigs. Regarding the transplantation pathways, the left branch of the portal vein inside the liver was superior to the jugular vein pathway. Thus, hPMSCs transplantation through the portal vein by B-ultrasonography may represent a superior approach for treating liver diseases.

## Background

Acute liver failure (ALF) is defined as the rapid development of severe acute liver injury with impaired synthetic function and hepatic encephalopathy in the absence of pre-existing liver diseases. The disease carries a high morbidity and mortality. Orthotopic liver transplantation is the most effective therapy for ALF, but it is highly intrusive, irreversible, and limited by a shortage of donor organs, high expense, and the necessity of lifelong immunosuppressive treatments [1,2]. Stem cell-based therapy, a promising alternative approach, is currently a focus of research worldwide [3,4]. Hepatocyte transplantation has been successfully reported in experimental animals as well as in some clinical human studies [5,6]. However, this procedure, which requires a large number of functional hepatocytes, is also restricted by the lack of available organs for cell isolation [7]. Novel cell sources are therefore needed to develop and improve the accessibility of cell-based therapies in hepatology. The preeminent candidate cells for this purpose are mesenchymal stem cells (MSCs), which can be obtained from various tissues, such as bone marrow (BM), muscle, tooth, periodontal ligament, amniotic fluid (AF), scalp tissue, dermis, placenta, adipose tissue (AT), and umbilical cord blood (UCB) [8-18]. Furthermore, isolated MSCs are multipotent and can differentiate into multiple lineage cell types including mesodermal cell lineages such as osteoblast, adipocyte, chondroblast, myocyte, and cardiomyocyte, as well as non-mesodermal cells such as hepatocyte and neurocyte [19]. Besides, MSCs have been demonstrated to be less immunogenic and can induce tolerance upon transplantation [20]. Due to their self-renewal capacity, multilineage differentiation potential and immunosuppressive qualities, MSCs are accepted as the most suitable source for cell-based therapy for liver diseases. Recently, their therapeutic potential in the treatment of liver injury has been evaluated. MSCs exhibited the highest potential for liver regeneration compared with other BM cell subpopulations in an animal model of hepatic injury [21]. Studies have demonstrated the capacity of bone marrow-derived mesenchymal stem cells (BM-MSCs) to differentiate into hepatocytes when directly xenografted to allylalcohol (AA)-treated rat liver [22]. Additionally, Jung *et al. *reported that human umbilical cord blood-derived mesenchymal stem cells (hUCB-MSCs) were able to ameliorate liver fibrosis in a carbon tetrachloride (CCl_4_)-induced cirrhotic rat model [23]. MSCs from human umbilical cord/adipose tissue also have the potential to improve liver function in mice with liver injury [17,24,25]. Another promising source of MSCs is the placenta. MSCs from human placenta (hPMSCs) display characteristics similar to those of MSCs from bone marrow, but enjoy several advantages [26]. They are free of ethical concerns, non-invasively accessible, abundant, and strongly immunosuppressive [27-29]. Placenta-derived cells also display multilineage differentiation potential and can be differentiated into hepatocyte-like cells *in vitro *[30]. Moreover, hPMSCs exert an anti-fibrotic effect in a rat model of the CCl_4_-injured liver [31], and transplanted hPMSCs also ameliorate carbon tetrachloride-induced liver cirrhosis in mouse.

However, the therapeutic potential of hPMSCs in a large animal model of hepatic injury remains largely unexplored. In the present study, we isolated MSCs from human placentas and characterized their morphology, phenotypic profiles, growth potency, and capacity to differentiate into various lineages. MSCs were transplanted directly into pigs with acute liver failure induced by G-galactosamine (GalN) via two routes: the jugular or portal veins. The intra-portal cell transplantation procedure was guided by B-ultrasonography. After transplantation, their capacity to overcome hepatic injury was investigated.

## Methods

### Isolation and culturing of human placental mesenchymal stem cells (hPMSCs)

Placentas were obtained from donors at the Hangzhou Red Cross Hospital in China after informed consent had been obtained. The study used a protocol approved by the Research Ethics Committee of the First Affiliated Hospital, School of Medicine, Zhejiang University.

The placental tissue was washed with preheated phosphate buffered saline (PBS, pH 7.2 ± 0.1, GenomSciences, Hangzhou, China), minced, and digested using 0.1% (w/v) collagenase type IV (Invitrogen, USA) at 37°C for 30 min. Recovered cells and digested cell debris were filtered through a 100-μm cell strainer. Mononuclear cells (MNCs) obtained by lymphocyte isolation (GE Healthcare, Ficoll-Pague™ PLUS, Sweden) were cultured in special medium (MesenCult^® ^Human Basal Medium plus MesenCult^® ^Human Supplement, STEMCELL Technologies Inc., Vancouver, Canada) and adjusted to 2 × 10^6 ^cells in T25-cm^2 ^tissue culture flasks (Nunc Flasks Nunclon™Δ with Filter Cap, Denmark) maintained in an incubator at 37°C in a humidified atmosphere with 5% (v/v) CO_2_. Approximately 4 to 6 days later, many colonies had formed. After achieving 60-70% confluence, adherent cells were trypsinized using 0.25% (w/v) trypsin/EDTA (Invitrogen, Carlsbad, CA, USA) and re-plated at a 1:3 dilution. Placenta-derived cells were cultured continuously under the same conditions.

### Surface antigen expression

Culture-expanded cells (passages 3 to 6) were washed with PBS-containing 0.5% (w/v) bovine serum albumin (BSA) and their concentration adjusted to 1 × 10^6 ^cells/100 μL. Their surface antigen expression was evaluated by incubating with anti-human antibodies against CD90-phycoerythrin (PE), CD13-PE, CD133-PE, CD31- allophycocyanin (APC), CD29-APC, CD73-APC, CD105-APC, CD79B-fluorescein isothiocyanate (FITC), CD34-FITC, CD166-FITC, and CD45-TRI COLOR (PE-Cy5- PC5) (eBioscience, Inc., San Diego, CA, USA). After being labeled with antibodies in the dark at room temperature for 20 min, cells were washed twice with PBS. Two-color flow cytometry was conducted using a Beckman Coulter Cytomics FC 500 MPL (Beckman Coulter, Inc., Los Angeles, CA, USA), and the data were analyzed using MXP software.

### Differentiation studies

#### Adipogenic and osteogenic differentiation

Adherent cells derived from placenta (passages 2 to 6) were plated at a density of 2 × 10^3^/well on six-well multidishes (Nunclon™Δ Surface, Denmark) and cultured in special conditional adipogenic medium (MesenCult^® ^Human Adipogenic Suppl, STEMCELL Technologies, Inc., Canada) as per the manufacturer's protocol. After 4 weeks, adipogenesis was confirmed by oil red O staining (Sinopharm Chemical Reagent Co., Ltd, Shanghai, China) and lipoprotein lipase (LPL) mRNA detection. Human intraoperative abandoned adipose tissue and hPMSCs cultured in normal growth medium served as controls.

MSCs from placenta (passages 2 to 6) were plated at a density of 2 × 10^3^/well in six- well plates (Nunclon™Δ Surface, Denmark) and expanded in special conditional osteogenic medium (OriCell™ hMSC Osteogenic Differentiation Medium, Cyagen Biosciences, Guangzhou, China) and media was changed twice per week. After 4 weeks, osteogenic differentiation was evaluated by Alizarin Red S staining (Genmed Scientifics Inc., USA, Shanghai, China) and detection of osteopontin (OPN) mRNA. hPMSCs cultured in normal growth medium and immortalized human fetal osteoblastic cells (hFOB 1.19 cell line, Cell Bank of the Chinese Academy of Sciences, Shanghai, China) served as controls.

#### Hepatic differentiation

Cultured placental cells (1 × 10^4^/mL; passages 2 to 6) were plated in T25-cm^2 ^tissue culture flasks (Nunc Flasks Nunclon™Δ with Filter Cap, Denmark) and four-well chamber slides (Nunc Lab-Tek™ Chamber Slide™ System, Denmark) with LG-DMEM (Gibco/Invitrogen, USA) containing 10% (v/v) FBS (Gibco/Invitrogen, USA) for 2 days. Hepatogenic induction was conducted as follows. The medium was changed to IMDM supplemented with 20 ng/mL epidermal growth factor (EGF) and10 ng/mL fibroblast growth factor (FGF) for 2 days. The cells were then treated with IMDM with 20 ng/mL hepatocyte growth factor (HGF), 10 ng/mL FGF, and 0.61 g/L niacin amide and 1% (v/v) insulin-transferrin-selenium (ITS) premix (Sigma-Aldrich, St Louis, MO, USA) for 10 days; the medium was changed every 3 days. Finally, cells were maintained in IMDM containing 20 ng/mL oncostatin M (OSM; St Louis, MO, USA), 1 μmol/L dexamethasone (Dex; Sigma-Aldrich, St Louis, MO, USA), and 1% (v/v) ITS premix for 10 days. For the next few days, cells were cultured with HEPATOZYME-SFM (Gibco/Invitrogen, USA). Cells cultured with low glucose DMEM (LG-DMEM) containing 10% (v/v) FBS served as controls. Albumin (ALB), alpha-fetoprotein (AFP), cytokeratin 18 (CK18), and cytokeratin 19 (CK19) levels were determined by immunohistochemistry and RT-PCR; and urea synthesis, low-density lipoprotein (LDL) uptake, glycogen storage, and cytochrome P450(CYP450) enzymatic activity were assayed to evaluate hepatogenic induction on days 7, 14, 21, 28, and 35 after induction.

### Immunocytochemistry

Cell samples were fixed in 4% (v/v) paraformaldehyde solution at room temperature for 15 min. After being treated with 0.3% (v/v) hydrogen peroxide (H_2_O_2_) and blocked in 5% (m/v) BSA, cells were incubated with diluted primary antibody against anti-human CK18 (2 mg/mL, 1:400; Abcam, UK), human AFP (1:200; Abcam, UK), human CK19 (1:400; Abcam, UK), and human ALB (5 mg/mL, 1:500; Abcam, UK), according to the manufacturer's instructions. The primary antibody was detected using horseradish peroxidase-conjugated anti-rabbit IgG (1 mg/mL, 1:1,000; Abcam, UK) antibody followed by incubation with diaminobenzidine tetrahydrochloride solution (DAB kit, Vector Labs, Burlingame, CA, USA). Cultured human hepatocytes served as positive controls.

### Reverse transcriptase-polymerase chain reaction (RT-PCR)

Total RNA of cells was extracted with the Trizol reagent (Invitrogen, USA). The quantity and purity of RNA were estimated by reading absorbance at 260 and 280 nm. cDNA was synthesized using oligo dT primers with the Improm-II™ Reverse Transcription System (Promega, Madison, WI, USA) according to the manufacturer's instructions. The primers for the target products were designed as in Table 1. Polymerase chain reactions (PCR) were carried out in a PCR thermal cycler (Thermo Hybaid, Waltham, MA, USA). PCR products were electrophoresed on a 1.2% (m/v) agarose gel containing 0.5 μg/mL ethidium bromide for nucleic acid visualization under UV light.

**Table 1:** Primers and conditions used for RT-PCR

Primer	Sequence	Amplicon size (bp)	Cycle
ALB	5'- CACAGTTGCAACTCTTCGTGAAAC-3' 5'- AGCAGTGCACATCACATCAACC-3	154	94°C/30 s; 55°C/40 s; 72°C/60 s
CK18	5'- CACCGTCGTCCGCAAAGCCT-3' 5'- CCTGCCAGACCCCCGGCTAT-3	269	94°C/30 s; 58°C/40 s; 72°C/60 s
CK19	5'-AGGTGGATTCCGCTCCGGGCA-3' 5'- ATCTTCCTGTCCCTCGAGCA-3'	460	94°C/60 s; 64°C/60 s; 72°C/120 s
AFP	5'- GCATGTGCAGTAATGAAAAAT-3' 5'- GAACAAAACTTGCCAAGAAGAT-3'	400	94°C/30 s; 50°C/40 s; 72°C/60 s
β-actin	5'- GAGCGGGAAATCGTGCGTGACATT-3' 5'- GATGGAGTTGAAGGTAGTTTCGTG-3'	234	94°C/30 s; 58°C/40 s; 72°C/60 s
LPL	5'-ATGGAGAGCAAAGCCCTGCTC-3' 5'-TACAGGGCGGCCACAAGTTTT-3'	298	94°C/30 s; 56°C/30 s; 72°C/60 s
OPN	5'-CTAGGCATCACCTGTGCCATACC-3' 5'-CTAGGCATCACCTGTGCCATACC-3'	330	94°C/30 s; 55°C/45 s; 72°C/60 s

### Glycogen storage staining

Primary human hepatocytes were obtained from the Key Lab of Combined Multi-organ Transplantation, Ministry of Public Health, P. R. China.

Undifferentiated and differentiated hPMSCs and primary human hepatocytes (5 × 10^5^/well) were plated in six-well plates and cultured in either LG-DMEM supplemented with 10% (v/v) FBS or HEPATOZYME-SFM (all from Gibco/Invitrogen, USA). Cells were stained with the periodic acid-Schiff (PAS) staining kit according to the manufacturer's protocol (Genmed Scientifics Inc., Shanghai, China).

### Low-density lipoprotein (LDL) uptake assay

Undifferentiated and differentiated hPMSCs and primary human hepatocytes (5 × 10^5^/well) incubated in DMEM containing 10% (v/v) FBS were labeled with DiI-LDL (1,19-dioctadecyl-3,3,39,39-tetramethylllindocarbocyane-low-density lipoprotein, Molecular Probes, Invitrogen, US) at 10 μg/mL for 24 h at 37°C. Cells were harvested, washed with cold PBS, and then stained according to the manufacturer's protocol. Uptake was subsequently evaluated by fluorescence microscopy (Olympus IX81, Japan).

### Urea assay

Undifferentiated and differentiated hPMSCs and primary human hepatocytes (5 × 10^5^/well) were cultured for 24 h in HepatoZYME-SFM (serum-free, no phenol red, Gibco^®^, Invitrogen™, USA) supplemented with 0.9 mmol/L ammonium chloride (NH_4_Cl, Bio Basic Inc., NY, USA). Supernatants were collected, centrifuged, and urea levels were measured in 96-well plates (Nunclon™Δ Surface, Denmark) at 430 nm by a spectrophotometer (Beckman Coulter^® ^Multimode Detector DTX880, Beckman Coulter, Inc.), according to the instructions of the QuantiChrom™ urea assay kit (DIUR-500, BioAssay Systems, CA, USA).

### Determination of cytochrome P450 activity

Undifferentiated and differentiated hPMSCs and primary human hepatocytes (5 × 10^5^/well) were plated in six-well plates and cultured in either LG-DMEM supplemented with 10% (v/v) FBS or HEPATOZYME-SFM (all from Gibco/Invitrogen, USA), both with 10 μM rifampicin for 48 h. The media were then changed to IMDM containing a luminogenic CYP substrate (luciferin-PFBE, 50 μM, 1:40 dilution) for 24 h. Intracellular CYP enzyme activities were assessed by assaying luciferin production, according to the manufacturer's instructions (P450-Glo™ CYP3A4 assay with leciferin-PFBE, Promega, USA). Luciferase activity was expressed as relative luminescence units (RLU).

### hPMSCs irradiation

hPMSCs from the placenta (passages 3 to 6) were seeded in T175-cm^2 ^tissue culture flasks (Nunc Flasks Nunclon™Δ with Filter Cap, Denmark). After achieving 70-80% confluence, cells were irradiated with 30 Gy at room temperature using six MV X-rays (500 Mu/min, total of 2890 Mu) from a Varian 23EX Linear Accelerator.

### Cell-viability assay

The lethal effect of irradiation on hPMSCs was determined using an MTT (3-(4,5-dimethylthiazol-2-yl)-2,5-diphenyltetrazolium bromide) assay. Each group of cells was seeded at a density of 3000 cells per well in 96-well plates (Nunclon™Δ Surface, Denmark). After irradiation for 0, 24, 48, 72, 96, 120, or 144 h, 20 μL of MTT reagent (5 mg/mL, Sigma-Aldrich) was added to each well, and cells were incubated for 4 h at 37°C and 5% (v/v) CO_2_. The formazan complex was dissolved in 150 μL dimethylsulphoxide (DMSO, Sigma-Aldrich), and absorbance at 490 nm was measured using a spectrophotometer (Beckman Coulter^® ^Multimode Detector DTX880, Beckman Coulter, Inc.). Experiments were performed in triplicate and repeated at least three times. In cell growth curves, the optical density (OD) values were plotted as a function of elapsed time.

### Cell-cycle and apoptosis analysis

The effect of irradiation on cell-cycle progression and apoptosis of hPMSCs was investigated by flow cytometry (Beckman Coulter, Inc.). Cell-cycle and apoptosis analyses were carried out at 0 and 6 h and at 3 and 7 days after irradiation treatment. For cell-cycle studies, cells were fixed with pre-cooled absolute ethanol (-20°C) and finally stained using a cell-cycle staining kit (MultiSciences, Hangzhou, China), according to the manufacturer's protocol. The distribution of cell-cycle phases with different DNA contents was determined by flow cytometry and analyzed using multicycle ANOVA analysis software. Cell-apoptosis analysis was carried out by flow cytometry using the Annexin V/PI apoptosis kit (MultiSciences, Hangzhou, China). The percentages of apoptotic cells were analyzed using MXP cytometer software. Experiments were repeated three times.

### D-galactosamine-induced acute liver-failure (ALF) model

Male Chinese experimental miniature pigs weighing 10-12 kg were obtained from Beijing Agriculture University (Beijing, China). Animal procedures were approved by the Animal Ethics Committee of Zhejiang University. All pigs were housed in an air-conditioned room with a 12 h dark/light cycle and received humane care for the duration of the study. For catheterization through the external jugular vein, ketalar (0.2 mL/kg) (Fujian Gutian Pharmaceutical Co., Ltd., Fujian, China) was injected intramuscularly to induce sedation. An auricular vein was secured and 2.5 mg/kg/h diprivan (AstraZeneca, Caponago, Italy) administered to achieve continuous anaesthesia. Catheterization was performed as described previously [32]. Six hours after the operation, acute liver injury in the pigs was induced by administration of D-galactosamine (GalN, Hanhong Chemical Co., Ltd, Shanghai, China) in 5% (w/v) glucose (pH 6.8) at a dose of 1.5 g/kg body weight via a venous catheter. Ultimately, all pigs without cell transplantation died due to GalN-induced hepatic failure, making sacrifice unnecessary. GalN-induced liver injury was identified histologically by a trained pathologist. Animals were considered to be dead when their heartbeat and breathing ceased and blood pressure could not be measured.

### Experimental groups and cell transplantation

Twenty-four ALF pigs were randomly divided into four groups (*n *= 6 each): Group I, ALF pigs without cell transplantation; Group II, hPMSCs transplantation via the jugular venous catheter; Group III, X-ray-treated hPMSCs transplantation via the portal vein; and Group IV, hPMSCs transplantation via the portal vein.

Eighteen hours after GalN administration, hPMSCs (passages 3 to 6; 1.0 × 10^8 ^in 5 mL saline) were directly transplanted into the pigs via the jugular venous catheter or the portal vein. For intra-portal cell transplantation, an 18 G PTC needle (Hakko Co., Chikuma-Shi, Japan) with a guide wire was inserted into the left branch of the portal vein inside the liver. The procedure was performed under general anesthesia and guided by color ultrasound (Sequoia 512, Siemens, Germany). By injecting contrast medium SonoVue (Bracco, Milan, Italy), the transplanted cells were seen to be distributed throughout the liver. When the guide wire was withdrawn, blood flow confirmed that the portal vein had been accessed. Then, the hPMSCs preparation was directly and slowly infused into the liver via the portal vein over about 5 min. For jugular vein cell transplantation, the same number and volume of hPMSCs were infused into the jugular vein via a venous catheter. All transplanted pigs were immunosuppressed by daily administration of dexamethasone (Hubei Tianyao Pharmaceutical Co., Ltd, Xiangfan, Hubei) (10 mg/kg/d) during the first week after the transplantation. Blood samples were collected for biochemical analysis at the following time points: pre-and post-GalN administration (18 h), 1 to 7 days after cell infusion, then every 2 weeks from the second week until death. Liver samples from surviving animals were harvested every 2 weeks post-transplantation using an automatic biopsy needle, and the livers of dead animals were directly removed by dissection. These liver tissues were immediately stored at -80°C for molecular detection or fixed in 10% (v/v) formalin for histological and immunohistochemical analysis.

### Biochemical analysis

Blood samples were obtained from each pig and centrifuged for 10 min at 1751 × *g *(Sorvall^® ^Biofuge Stratos, Thermo, Germany) and serum was collected. Concentrations of liver injury markers, such as alanine aminotransferase (ALT), aspartate aminotransferase (AST), alkaline phosphatase (ALP), total bilirubin(TBIL), and total bile acids (TBA) were measured with an automated biochemical analyzer (Abbott Aeroset, Abbott Laboratories, Chicago, IL, USA). All samples were run in triplicate.

### Cytokine level measurement

Supernatants of hPMSCs (passages 3 to 6), hPMSCs co-cultured with 1 × 10^6 ^lymphocytes from healthy donors for 24 h and serum from model pigs, were collected, centrifuged, and leukemia inhibitory factor (LIF) levels were analyzed using a human LIF platinum ELISA Kit (eBioscience, Inc., San Diego, CA, USA). Tests were performed according to the manufacturer's protocol and every sample was run in duplicate. LIF concentration (pg/mL) was determined by comparison with the standard curve.

hPMSCs (1 × 10^6^) and their supernatants were co-cultured with PBMC (1 × 10^6^) for 24 h. Interferon-γ (IFN-γ) secretion was then assayed by ELISpot, according to the manufacturer's instructions (human IFN-γ ELISpot PRO kit, MABTECH AB, Sweden). The resulting spots were counted and analyzed using a computer-assisted AID ELISPOT Reader System (Immun Spot^®^, Cellular Technology Ltd.).

### Pathology and immunohistochemistry

Pig livers were harvested at the indicated time points after cell transplantation, fixed in 10% (v/v) formalin, and embedded in paraffin. Sections (5-μm thickness) were deparaffinized, rehydrated, and stained with hematoxylin and eosin (H&E) for routine histology. For immunohistochemical evaluation, the sections were heated in citrate buffer (0.02 mol/L, pH 5.8) for antigen retrieval. Endogenous peroxidase was prevented by immersion in a 0.3% (v/v) hydrogen peroxide (H_2_O_2_) in methanol bath for 15 min. After washing, non-specific binding was blocked with 5% (m/v) bovine serum albumin in PBS. Sections were then incubated with diluted primary antibody against anti-human ALB (5 mg/mL, 1:5,000; Abcam, UK), human AFP (1:250; Abcam, UK), and human CK18 (1 mg/mL, 1:200; Abcam, UK), according to the manufacturer's instructions. Detection of primary antibody was performed using horseradish peroxidase-conjugated secondary antibodies (1 mg/mL, 1:1,000; Abcam, UK). Peroxidase activity was revealed by a 3 to 5 min exposure to diaminobenzidine tetrahydrochloride solution (DAB kit, Vector Labs). The sections were then washed, counterstained with hematoxylin for one minute, mounted, and observed under a light microscope (TE2000, Nikon, Japan). Normal human tissue obtained under informed consent served as positive controls.

### Detection of human *alu *sequences by PCR

PCR for human *alu *sequences was performed to confirm the presence of transplanted hPMSC-derived cells in recipient pig livers. Total DNA of liver samples was extracted with a DNeasy^® ^Blood & Tissue Kit (Qiagen, Hilden, Germany), according to the manufacturer's instructions. The primers used were 5'-CTGGGCGACAGAACGAGATTCTAT-3' and 5'-CTCACTACTTGGTGACAGGTTCA-3'; samples were incubated at 94°C for 2 min and then amplified for 35 cycles of denaturation for 30 s at 94°C, annealing for 30 s at 60°C, and extension for 59 s at 72°C. The PCR products were analyzed by resolution on a 1.2% (w/v) agarose gel and visualized by ethidium bromide staining. Images were captured using a gel documentation system (Syngene GBox-HR Gel Doc System, UK).

### Statistical analysis

Data were presented as the mean ± standard deviation (SD). Differences in serum levels of biochemical parameters were analyzed using a paired-samples *t*-test. Statistical evaluations of cell viability, cell-cycle distribution, and percentage apoptosis, urea synthesis, CYP-450 enzymatic activity, LIF, and IFN-γ secretion were performed using a *t*-test. Animal survival was analyzed using the Kaplan-Meier log rank method. Values of *P *< 0.05 were considered statistically significant. Data were analyzed with SPSS ver. 16.0 statistical software (SPSS, Inc., Chicago, IL, USA).

## Results

### Morphology and phenotype profiles of hPMSCs

**Figure 1:**
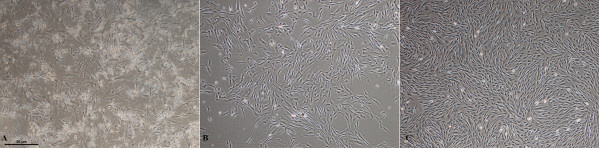
**hPMSCs morphology (4×)**. (**A**) Three days after isolation from the placenta before changing media; (**B**) four days after isolation from the placenta after changing media; (**C**) four weeks after isolation (passage 6).

Primary adherent monolayer placental cells formed many colonies after 4 to 6 days and exhibited a fibroblastic morphology. They were subcultured every 3 to 4 days (Figure 1).

**Figure 2:**
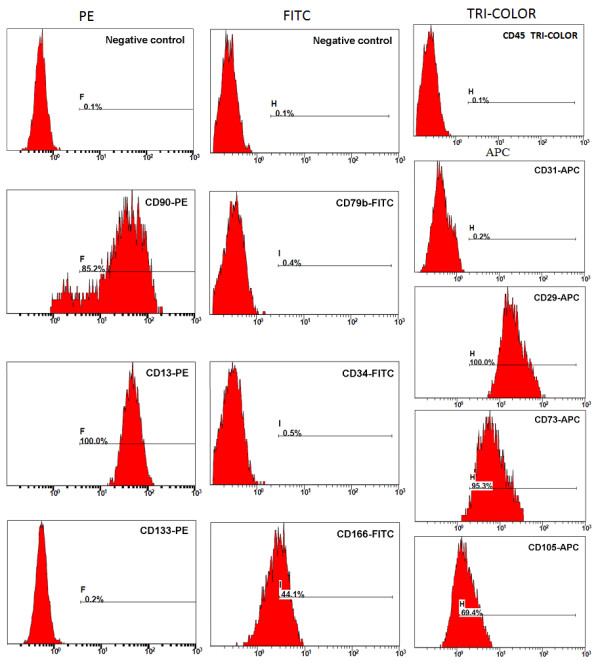
**Phenotype profile of adherent cells obtained from placentas**. Immunophenotype of hPMSCs determined by flow cytometry at passage three using labeled antibodies specific for the indicated human surface antigens or negative controls.

The immunophenotype of culture-expanded cells (passages 3 to 6) was analyzed by flow cytometry, which revealed that the cells expressed high levels of CD29 (100%), CD73 (95.3%), CD13 (100%), and CD90 (85.2%), moderate levels of CD105 (69.4%) and CD166 (44.1%), but expressed almost no hematopoietic cell markers such as CD45 (0.1%), CD34 (0.5%), and CD133 (0.2%). CD31 and CD79b were expressed in only 0.2 and 0.4% of the population, respectively (Figure 2). These findings are consistent with the general description of the phenotype profile of classical hPMSCs, confirming that the cells were of the MSC lineage, but not hematopoietic cells.

### Adipogenic differentiation potential of hPMSCs

**Figure 3:**
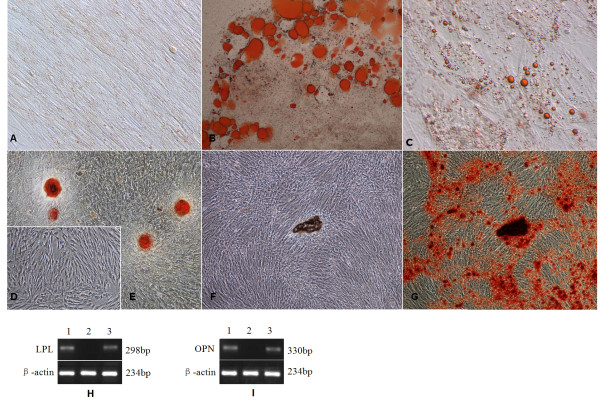
**Adipogenic (A-C, H) and osteogenic (D-G, I) differentiation of hPMSCs**. (**A**) hPMSCs maintained in normal growth medium (10×); (**B**) human adipose tissue as a positive control (10×); (**C**) hPMSCs that had undergone adipogenic differentiation (accumulation of lipid droplets shown by red staining) (20×); (**D**) hPMSCs maintained in normal growth medium; (**E**) osteoblast as a positive control(4×); (**F, G**) hPMSCs that had undergone osteogenic differentiation (bone nodules formed), before and after staining (4×). (**H, I) **Representative RT-PCR analyses of hPMSCs cultured in adipogenic and osteogenic medium for 28 days. No LPL expression was found in hPMSCs maintained in regular medium (H, lane 2). After adipogenic induction, hPMSCs expressed LPL mRNA (H, lane 1). Human adipose tissue expressed LPL mRNA (H, lane 3). No OPN expression was found in hPMSCs maintained in growth medium (I, lane 2). After osteogenic induction, hPMSCs expressed OPN mRNA (I, lane 1). Osteoblasts expressed OPN mRNA (I, lane 3). LPL, lipoprotein lipase; OPN, osteopontin.

hPMSCs were cultured for 4 weeks in adipogenic medium, and adipogenic differentiation was assessed by oil red O staining and LPL mRNA detection. Differentiated hPMSCs formed intracytoplasmic lipid droplets and could be identified by oil red O staining, whereas hPMSCs cultured in normal growth medium had no lipid droplets and exhibited negative staining. Human adipose tissue (positive control) exhibited large lipid vesicles (Figure 3A, B, and 3C). Adipogenic differentiation of hPMSCs was further confirmed by expression of adipocyte-specific genes (LPL), as determined by RT-PCR (Figure 3H).

### Osteogenic differentiation potential of hPMSCs

hPMSCs were cultured for 4 weeks in osteogenic medium, and osteogenic differentiation was assessed by Alizarin Red S staining and bone-specific gene (OPN) mRNA detection. Differentiated hPMSCs and osteoblast formed bone nodules and stained positive, whereas hPMSCs cultured in normal growth medium showed no positive staining (Figure 3D-G). Osteogenic differentiation of hPMSCs was further confirmed by OPN expression, as determined by RT-PCR (Figure 3I).

### Hepatic differentiation potential of hPMSCs

**Figure 4:**
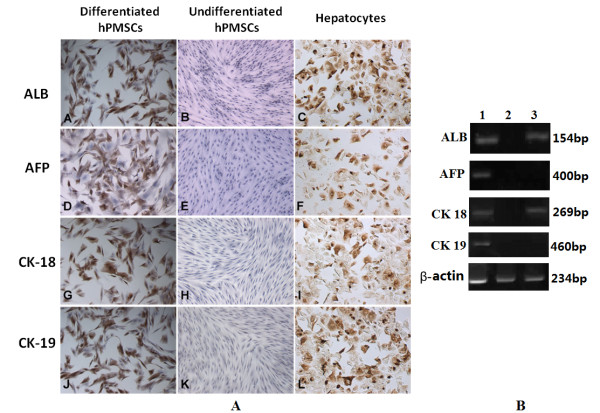
**Hepatic differentiation of hPMSCs**. (**A**) Immunocytochemistry analysis of hepatically differentiated or undifferentiated hPMSCs. (**A**, **D**, **G**, and **J**) Staining for the hepatic markers ALB, AFP, CK18, and CK19; a large number of cells had a light-brown coloration. (**B**, **E**, **H**, and **K**) hPMSCs cultured in growth medium did not display positive immunocytochemical staining. (**C**, **F**, **I**, and **L**) Efficiency of immunocytochemical staining was tested on cultured hepatocytes as positive controls (10×). (B) RT-PCR analysis of hepatic-specific gene expression in hepatically differentiated or undifferentiated hPMSCs. Lane 1, hPMSCs that had undergone hepatic differentiation; lane 2, hPMSCs cultured in growth medium; and lane 3, human liver tissue as a positive control.

hPMSCs at passage three were cultured in differentiation medium with hepatogenic supplements for 3 weeks. Differentiation efficiency was evaluated at the protein and mRNA levels. Immunocytochemistry revealed the presence of hepatic markers (ALB, AFP, CK18, CK19) in hPMSCs subjected to the hepatogenic differentiation protocol as well as in hepatocytes, but not in undifferentiated cells (Figure 4A). Similar results were obtained by RT-PCR analysis (Figure 4B), which revealed the expression of ALB, AFP, CK-18, and CK-19 mRNA by these differentiated cells. No amplification occurred in hPMSCs cultured in the absence of hepatogenic inducers.

**Figure 5:**
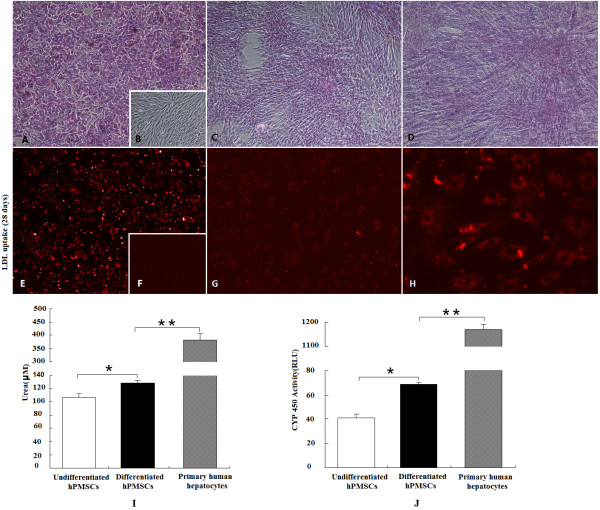
**Functional parameters of hepatocyte-like cells differentiated from hPMSCs**. (**A-D**): glycogen storage capacity. (**A**) Primary human hepatocytes as a positive control (10×); (**B**) hPMSCs cultured in normal growth medium did not display positive PAS staining (4×); (**C, D**) hPMSCs cultured in hepatogenic differentiation medium for 28 days were positively stained, revealing cytoplasmic glycogen accumulation (C, 4×; D, 10×). (**E-H**) LDL uptake. (**E**) Primary human hepatocytes served as a positive control (10×); (**F**) hPMSCs maintained in growth medium were stained negatively; (**G, H**) LDL uptake was detected in hepatocyte-like cells 21 days after differentiation (G, 10×; H, 20×). (**I**) urea synthesis assay. After hepatogenic differentiation culture for 28 days, urea levels in culture supernatants were significantly increased. (**J**) CYP-450 enzymatic activities were determined. After hepatogenic differentiation culture for 35 days, CYP-450 enzymatic activity was significantly higher. Data are mean ± *SD *(n = 4, *P < 0.05). But urea synthesis and CYP3A4 enzymatic activities of the hepatocyte-like cells derived from hPMSCs were significantly lower than those of the primary human hepatocytes (**P < 0.01).

Hepatocyte-like cells differentiated from hPMSCs exhibited characteristics typical of hepatocytes such as LDL uptake, glycogen storage, urea synthesis, and CYP-450 enzymatic activity, which were absent in undifferentiated MSCs (Figure 5). Primary human hepatocytes served as a positive control.

### Effect of X-ray irradiation on the viability and cell-cycle progression of hPMSCs

**Figure 6:**
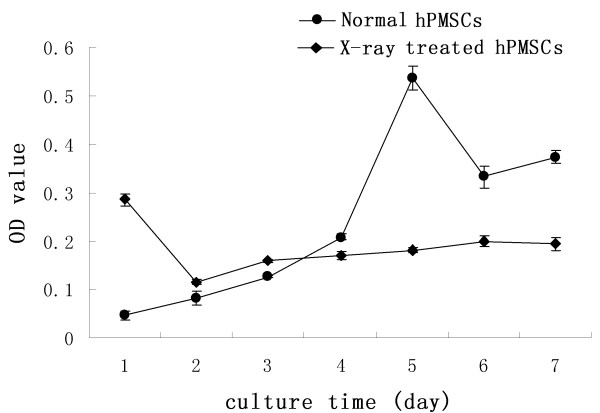
**Effects of X-ray irradiation on hPMSCs viability**. Cells were treated with X-rays for the times indicated, and their viability was measured by MTT assay. OD indicates optical density at 490 nm.

To investigate the effect of X-ray irradiation on the viability of hPMSCs, MTT assays were performed and a cell growth curve generated. No increase in cell numbers was found in hPMSCs treated with X-rays (Figure 6), suggesting a lethal effect. In contrast, a significant increase in cell number was observed in normal hPMSCs, which continued to increase until the plateau phase. These data suggest that X-ray irradiation can compromise the viability of hPMSCs.

**Figure 7:**
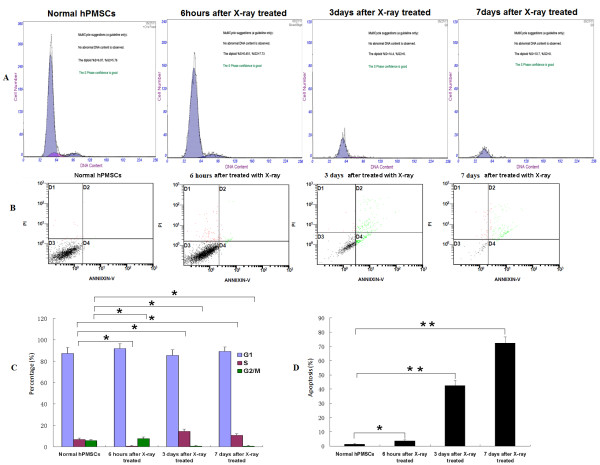
**Effects of X-ray irradiation on cell-cycle progression and apoptosis of hPMSCs**. Cells were irradiated with X-rays for the times indicated, and cell-cycle progression and apoptosis were analyzed by flow cytometry. (**A**) Representative cell-cycle profile of hPMSCs. (**B**) Cytograms of hPMSCs apoptosis assays. (**C**, **D**) Quantitative analyses of the FACS data. Data are presented as means ± SD of triplicate experiments; *P < 0.05, **P < 0.01.

To assess the effects of X-ray irradiation on cell-cycle progression and apoptosis, flow cytometry was utilized. Compared to normal hPMSCs (G_1_, 87.4%; S, 6.87%; G_2_/M, 5.8%), 89.3% of X-ray treated cells (7 days post-treatment) were in G_1 _phase, 10.7% in S phase, and 0% in G_2_/M phase (Figure 7). These data suggest that X-rays can arrest hPMSCs in G_1 _phase. Treatment with X-rays also resulted in significant apoptotic responses. The percentage of apoptotic cells was 1.26% in the normal hPMSCs group; 7 days after treatment with X-ray, it increased to 72.34%. These findings reveal that X-rays can significantly induce hPMSCs apoptosis. Taken together, our data suggest that X-ray irradiation dramatically blocked cell cycle progression and induced apoptosis of hPMSCs.

### Survival of ALF pigs transplanted with hPMSCs

**Figure 8:**
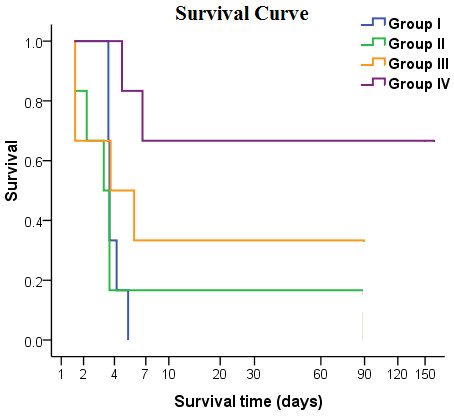
**Survival curves of the four groups**. Group I: injured, non-transplanted; Group II: injured, transplanted with normal hPMSCs via the jugular vein; Group III: injured, transplanted with X-ray-treated hPMSCs via the portal vein; Group IV: injured, transplanted with normal hPMSCs via the portal vein. The survival rate of Group IV was prolonged significantly compared with the other three groups (*P *< 0.05).

In Group I, all pigs died within 6 days (survival time, 94.7 ± 15.4 h); in Group II, all pigs died within 4 days (survival time, 67 ± 22.5 h) except one (sacrificed at 3 months); in Group III, four pigs died within 6 days (survival time, 75.8 ± 48.0 h) and two others were sacrificed at 3 months; in Group IV, two pigs died after 7 days (survival time, 135 ± 33.9 h) and four were sacrificed at 5 months. In Groups I to IV, 7-day survival rates were 0, 16.7, 33.3, and 66.7%, respectively. The survival of pigs in Group IV was prolonged significantly compared with the other three groups (*P *< 0.05; Figure 8).

### Restoration of liver functions in ALF pigs transplanted with hPMSCs

**Figure 9:**
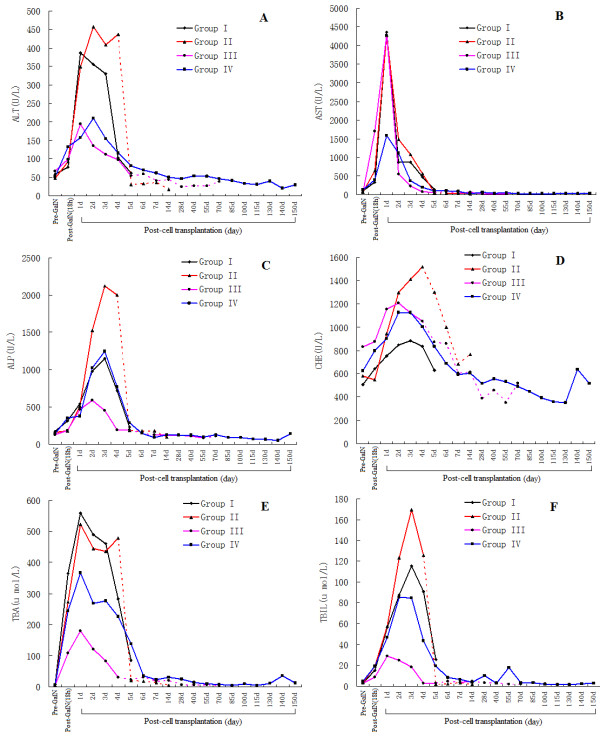
**Biochemical analyses**. (**A**) Alanine aminotransferase (ALT); (**B**) aspartate aminotransferase (AST); (**C**) alkaline phosphatase (ALP); (**D**) cholin esterase (CHE); (**E**) total bile acids (TBA); (**F**) total bilirubin (TBIL). Group I: injured, non-transplanted; Group II: injured, transplanted with normal hPMSCs via the jugular vein; Group III: injured, transplanted with X-ray-treated hPMSCs via the portal vein; Group IV: injured, transplanted with normal hPMSCs via the portal vein. Data are presented as means ± SD. The dotted line represents serum biochemical results of surviving pigs in Groups II and III.

Biochemical parameters (ALT, AST, ALP, CHE, TBIL, and TBA) were measured at selected time points to determine the restoration of liver functions in ALF pigs transplanted with hPMSCs (Figure 9). The serum levels of these factors in Groups I, II, and III were significantly increased 1 to 3 days after GalN-induced injury, and remained high until death. These parameters in Group IV pigs also increased initially but then decreased gradually from day 4 and were approaching normal levels one week after cell transplantation. The biochemical analyses revealed significant differences between pre- and post-GalN treatment in all four groups, suggesting that the ALF pig models were successfully generated. Also, in Group IV, the differences in these parameters were significant between days 7 and 2 post cell transplantation (*P *< 0.05), indicating that hPMSCs transplanted via the portal vein significantly recovered GalN-induced liver damage.

### Cytokine secretion and immunoregulation of transplanted hPMSCs

**Figure 10:**
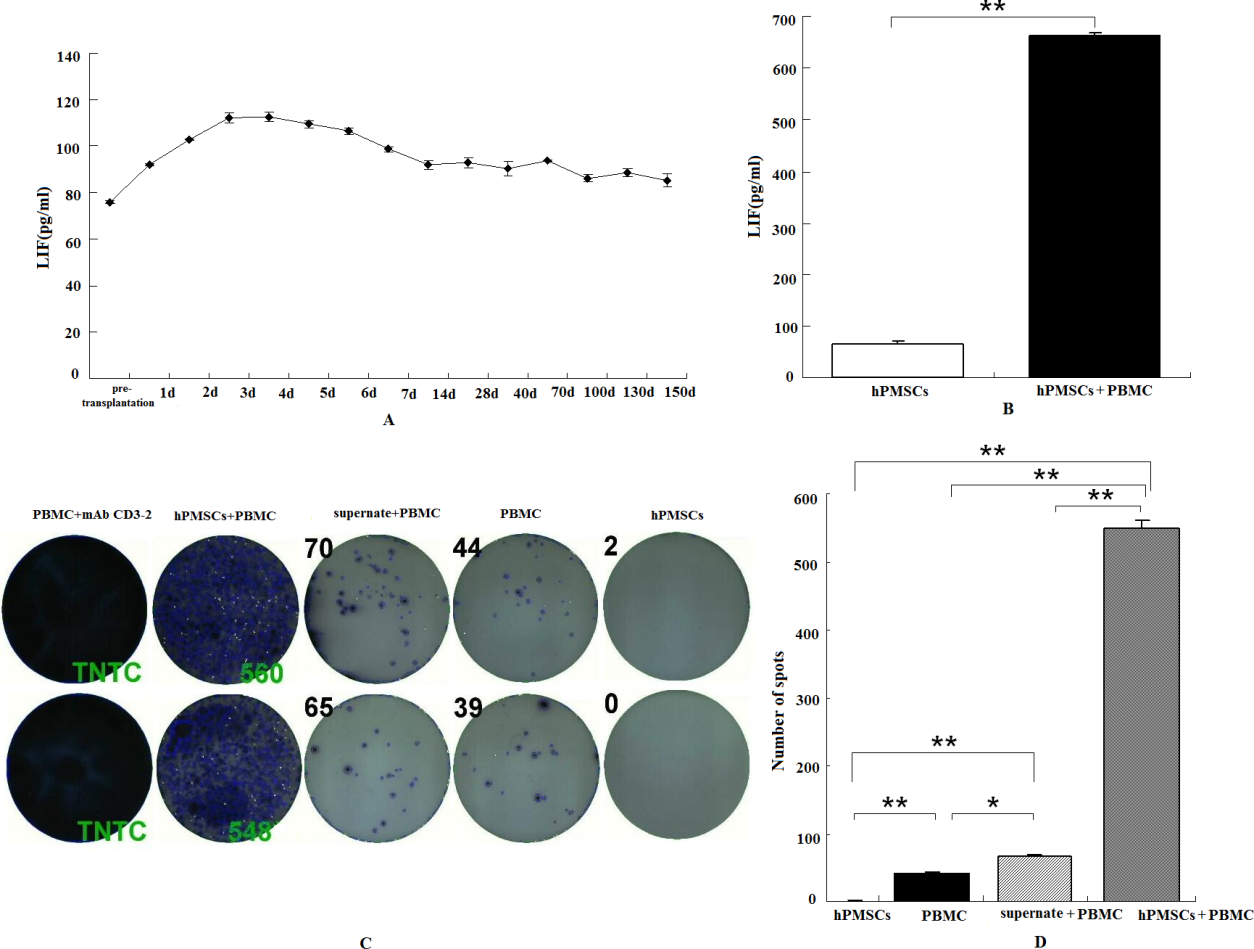
**Levels of human LIF and IFN-γ**. (**A**) LIF curve in the serum of Group IV pigs; (**B**) LIF was markedly increased after hPMSCs were co-cultured with PMBCs for 24 h. **P < 0.01; (**C, D**) hPMSCs stimulated PBMCs to secret IFN-γ. Each spot represents one human IFN-γ secreting cell. Spots were counted using ImmunoSpot ver. 5.0.3. The spots of the positive control were too numerous to count (TNTC). *P < 0.05, **P < 0.01.

To determine the immunoregulatory activity of hPMSCs, human LIF and IFN-γ were detected in the sera of model pigs and hPMSCs supernatants. In the four long-term survivors of Group IV, human LIF in the serum peaked on day 4 and then returned to normal (Figure 10A). The results of Groups I to III were irregular. After hPMSCs were co-cultured with PMBCs for 24 h, LIF exhibited a nine-fold increase (663.6 pg/mL) in hPMSCs/PBMCs as compared to hPMSCs alone.

ELISpot assay revealed that hPMSCs themselves did not secret IFN-γ, but they could stimulate PBMCs to secret IFN-γ (Figure 10B).

Therefore, hPMSCs may contribute to therapeutic effects in ALF models through immunoregulation.

### Histological features of the ALF pig livers transplanted with hPMSCs

**Figure 11:**
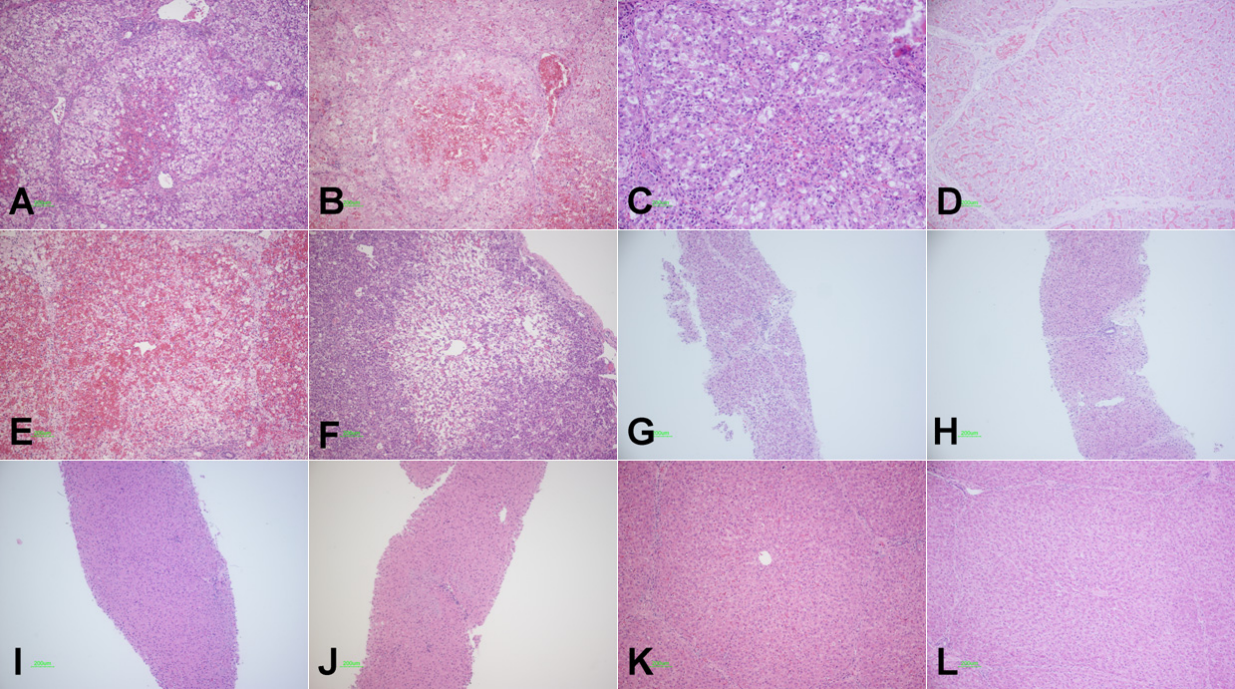
**H&E staining**. (**A**) Group I (day 4); (**B**) group II (day 1); (**C, D**) group III (days 4, 7); (**E-L**): group IV (days 2, 4, 7, 14, 28, 42, 150, and 155) (10×); (**G-J**) liver puncture biopsy specimens.

Livers from each group were collected at selected time points and H&E staining was performed to examine liver pathology. Galactosamine-induced liver injury in the control group (Group I) was indicated by large areas of inflammation, hepatocyte denaturation, and necrosis, as well as sinusoid congestion and hemorrhage (Figure 11A). Transplantation of hPMSCs via the portal vein (Group IV) had a beneficial effect on recovery of the GalN-injured liver (Figure 11E-L). A visible amelioration was found in Group IV 4 days after infusion of hPMSCs (Figure 11F); this was confirmed by milder damage and lighter lesions. Hemorrhage was markedly decreased. Hepatocyte denaturation and necrosis were also suppressed, accompanied by visible liver regeneration. Furthermore, these effects became more prominent over time. Hepatocyte denaturation and necrosis and hemorrhage disappeared beginning 7 days after transplantation, and the impaired hepatic lobule returned to a nearly normal architecture (Figure 11G-L). X-ray-treated hPMSCs also exerted a beneficial effect on recovery of the GalN-injured liver, promoting liver regeneration (Figure 11C) and lobular architecture recovery (Figure 11D). However, no significant improvement was seen in terms of hepatocyte denaturation and necrosis and hemorrhage (Figure 11C and 11D). No such amelioration was found in non-transplanted pigs (Group I) or in pigs transplanted with normal hPMSCs via the jugular vein (Group II) (Figure 11A and 11B).

### Fate of transplanted hPMSCs in ALF pig livers

**Figure 12:**
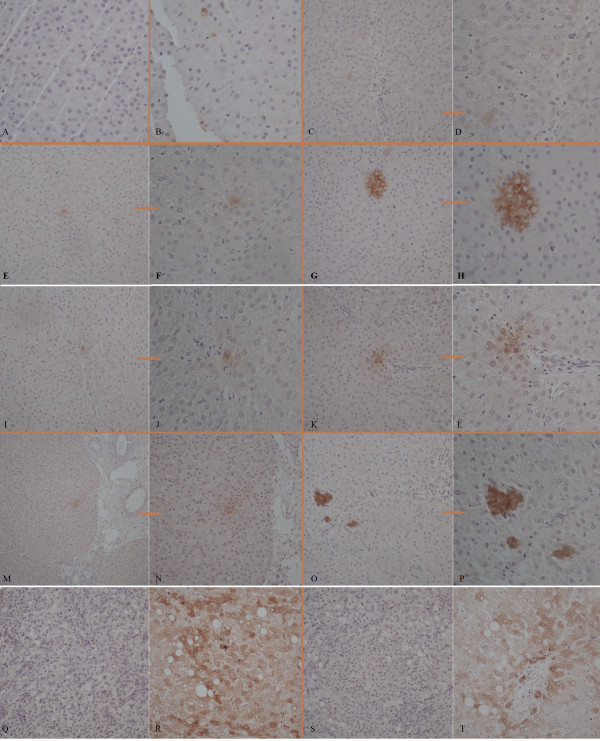
**Immunohistochemistry results of transplanted ALF pig liver samples**. (**A**) No ALB^+^cells were found in liver puncture biopsy samples 8 weeks after transplantation in Group IV (40×); (**B**) ALB^+ ^cells were detected 6 weeks after hPMSCs infusion in the specimen harvested using an automatic biopsy needle (40×); (**C-H**) ALB^+^cells were observed in three pigs at 5 months (**C**, **E**, **G**, 20×; **D**, **F**, **H**, 40×); (**I-P**) CK 18^+^cells were observed in three pigs at 5 months (**I**, **K**, **M**, **O**, 20×; **J**, **L**, **N**, **P**, 40×); (**Q, S**) non-transplanted pig livers served as negative controls for ALB (Q) and CK 18 (S) staining (20×); (**R, T**) sections of human liver served as positive controls for ALB (R) and CK 18 (T) staining (40×). It is noteworthy that almost all ALB^+ ^or CK18^+^cells were adjacent to vascular structures.

To investigate whether hPMSCs are capable of engrafting and undergoing hepatic differentiation *in vivo*, liver specimens taken from surviving animals (Group IV) were examined for the expression of the liver specific markers, ALB, AFP, and CK18, by immunohistochemistry of serial sections. As shown in Figure 12, ALB expression was only detected at the sixth week after hPMSCs infusion, showing single spotty positivity staining in the specimen harvested using an automatic biopsy needle. ALB^+^or CK 18^+^cells were observed in three of the four pigs sacrificed at 5 months, and a small cluster of cells with more intense staining was detected in one liver. However, no positive staining for CK18 was observed in any liver biopsy specimen. Most ALB^+ ^or CK18^+^cells were located in close proximity to vascular structures. Additionally, expression of human AFP, a marker of hepatic progenitor cells, was also investigated by immunohistochemistry. We were, however, unable to find even a single human AFP-expressing cell in hPMSCs-transplanted pigs (data not shown). Liver sections from Group I (non-transplanted pigs) were completely negative for ALB, AFP, and CK18 staining (Figure 12).

**Figure 13:**
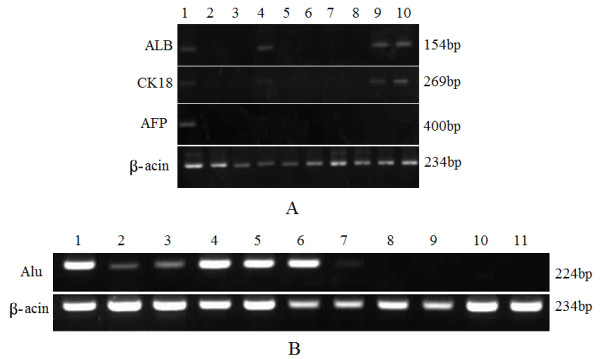
**Detection of human-specific hepatic markers by RT-PCR (A) and *alu *repeat sequences by PCR (B)**. (**A**): Lane 1, positive control (human liver for ALB and CK 18, hepatocellular carcinoma liver for AFP; lane 2: Group I as a negative control; lane 3 to 10, pig livers transplanted after 4, 6, 8, 12, 16, and 20 weeks and 5 months (double). Before 5 months, the samples were all obtained by liver biopsy puncture. Human ALB and CK18 were detected in the sixth week (lane 4) and the fifth month (lanes 9 and 10). (**B**): Lane 1, positive control (human DNA); lanes 2 to 6, Group IV (2, 4, 7, and 156 days after cell transplantation); lanes 7 and 8, Group III (2 and 4 days after cell transplantation); lanes 9 and 10, Group II (4 and 24 days after cell transplantation); lane 11, Group I (4 days after GalN infusion).

Similar results were obtained by RT-PCR analysis using human-specific primers. A 154-bp albumin band and a 269-bp CK18 band were detected in sixth-week liver biopsy specimens (in one of four pig samples) as well as three pigs sacrificed at 5 months (in three of four samples). Again, human AFP mRNA was not detectable by RT-PCR in the livers from hPMSCs-transplanted pigs, confirming the results obtained by immunohistochemistry. No expression of human albumin, AFP, or CK18 transcript was detected in non-transplanted pig livers (Figure 13A).

To identify transplanted human cells in recipient pigs, genomic DNA was extracted from recipient livers, and human-specific *alu *repeat sequences were amplified by PCR (Figure 13B). On the second day following cell injection, weak expression of human *alu *sequences was observed in Group IV (lane 2), which was enhanced on day 4 (lane 3). This human gene mark was expressed at a high level beginning on day 7 (lane 4), followed by continuous expression up to 156 days (lanes 5 and 6). Weak expression of this gene was also detected in Group III 1 day after cell transplantation (lane 7), but became undetectable beginning on day 3 (lane 8). There was no evidence of expression at any time point in Groups I and II (lanes 9, 10, and 11).

## Discussion

According to the International Society for Cellular Therapy (ISCT), MSCs are defined by their expression of CD105, CD73, and CD90 and lack of expression of CD45, CD34, CD14 or CD11b, CD79a or CD19, and HLA-DR. Additionally, they usually differentiate to osteoblasts and adipocytes *in vitro *[33]. These data were based on plastic-adherent cells isolated from BM and some other tissues. Our data revealed that besides having adipogenic and osteogenic differentiation potential *in vitro*, MSCs from the placenta expressed high levels of CD73, CD90, CD13, and CD29, but not hematopoietic cell markers such as CD45, CD34, and CD133. The different phenotype profiles of MSCs from the BM and placenta indicates the difference in their biological properties. Also, hPMSCs had hepatic differentiation potential *in vitro*, characterized by the expression of ALB, AFP, CK18, and CK19, production of urea, glycogen storage, and LDL uptake. MSCs from the placenta may be an excellent source for liver disease therapeutics because they are obtained easily, exhibit a wide variety of favorable properties, and can be expanded on a large scale [26,30,34,35].

Comparing with primary human hepatocytes, hepatocyte-like cells derived from hPMSCs showed much lower levels of LDL uptake and CYP-450 enzymatic activity. Since hepatocytes in intact liver are maintained in a three-dimensional environment and supported by surrounding cells such as Kupffer cells and liver endothelial cells, even primary hepatocytes will quickly lose activities during routine two-dimensional *in vitro *culture [36], it is therefore no surprise that the hPMSCs-derived hepatocyte-like cells cultured under similar conditions exhibited suboptimal hepatic activities. Nevertheless, our data clearly demonstrated the hepatogenic potential of hPMSCs that were employed for transplantation.

Currently, rodent models, including rat and mouse, are used to evaluate the potential of MSCs from the BM and placenta in repairing liver damage [37-41]. To explore the clinical value of hPMSCs, we used Chinese miniature pigs, a representative mammal, to establish a liver-failure model by D-galactosamine administration. The survival rate was significantly higher in the transplantation group than in the control group (66.7 *vs*. 0%). Additionally, the ALT, AST, ALP, CHE, TBIL, and TBA levels were significantly decreased in the transplantation group. To determine if hPMSCs moved to the injured pig livers had differentiated into hepatocyte-like cells, expression of hepatocyte-specific markers, such as ALB, AFP, and CK18, was investigated by immunohistochemistry and RT-PCR. Clusters or scattered cells positive for human albumin and CK18 were observed in recipient pig livers, particularly at 5 months after hPMSCs infusion. However, only positive staining for human ALB was observed in all liver biopsy specimens 6 weeks post-transplantation. RT-PCR analysis yielded similar results. In addition, AFP, a marker of immature liver progenitor cells, was detected by neither immunohistochemistry nor RT-PCR in livers from all hPMSCs recipient pigs. This may have been due to a limitation of the sampling method: because only a small quantity of biopsied liver samples was attainable, the probability of detecting colonized hPMSC-derived cells in these samples was low.

To verify that the observed effects were caused by transplanted hPMSCs but not the transplantation procedure *per se *or other processes, we further exposed hPMSCs to a lethal dose of X-rays (30 Gy) and transplanted them into Chinese miniature pigs as a control for transplanted cells. MTT and cell-cycle tests revealed that the viability and proliferation rate of hPMSCs after irradiation were significantly decreased; the percentage of G_2 _cells decreased from 5.76% to 0%. At the same time, the apoptosis rate of hPMSCs increased from 1.26% (no irradiation) to 3.7% (6 h after irradiation), 42.41% (3 days after irradiation), and 72.34% (7 days after irradiation). The survival rate of pigs receiving irradiated hPMSCs was significantly lower than that of pigs receiving non-irradiated hPMSCs (33.3% *vs*. 66.7%) but significantly higher than pigs without hPMSCs transplantation (33.3% *vs*. 0%). The following possible mechanisms may account for the observed effects of hPMSCs transplantation: (1) The transplanted hPMSCs may fuse with hepatocytes and initiate proliferation [42-44]. We found fewer ALB- and CK 18-positive cells in transplanted model pigs. Thus fusion of hPMSCs-derived hepatocyte-like cells with recipient hepatocytes cannot be excluded; (2) Homing of transplanted hPMSCs to the recipient liver which may differentiate into normal hepatocytes and result in replenishment of the hepatocyte reservoir [45-47]. Our data revealed that some cells expressed human ALB and CK 18 in recipient pig livers 5 months after MSCs transplantation; (3) Transplanted hPMSCs may support and activate liver progenitor cells, which then further initiate endogenous cell differentiation and proliferation [48,49]; (4) Transplanted hPMSCs may secrete multiple cytokines and growth factors that will inhibit inflammation and accelerate liver restoration [50-52]. Although hPMSCs proliferation was significantly decreased after irradiation, these cells may participate in the repair of damaged liver through pathways 3 and/or 4. In fact, our LIF and IFN-γ data provide evidence supporting the immunomodulatory roles of transplanted hPMSCs [53-56]. hPMSCs transplantation can contribute not only to hepatocyte repopulation, but also to the improvement of the internal liver environment.

The number of MSCs transplanted is a key factor, yet little is known about the optimum dose. Most studies have used doses of 2-10 million MSCs per kg in small-animal experiments [57-60]. Some researchers think that the greater the number of MSCs transplanted, the greater the therapeutic effect should achieve. In this study, a total dose of 1.0 × 10^8 ^hPMSCs in 5 mL saline was safe when infused into the liver within about 5 min. No embolism occurred, but the exact correlation between the number of cells transplanted and their therapeutic effect needs further investigation.

Choosing an appropriate MSC transplantation route is vital for cell survival, induction of cell differentiation, and restoration of liver function. Routine transplantation paths include those through the spleen, liver, belly cavity, and peripheral and portal veins, each of which has disadvantages. MSC transplantation through the spleen is an accepted route, but the transplanted cells usually grow in nodules and hence contribute little to the restoration of liver function. MSC transplantation by hepatic multi-site injection involves the risk of damaging important liver vessels, causing severe complications including hemorrhea, portal hypertension, and so on. MSC transplantation through the belly cavity is simple but also involves the risk of inducing inflammation and fever and causing peritonitis and adhesion in the belly cavity. We compared the effects of transplantation through peripheral (jugular) and portal veins and our data suggested that both transplantation routes were safe, with no portal vein thrombosis. Additionally, hPMSCs were well distributed in the liver after transplantation through the left branch of the portal vein under guidance of B-ultrasonography. The long-term survival rate in the portal vein transplantation group was significantly higher than that in the peripheral vein group (66.7% *vs*. 16.7%). A plausible mechanism may be the high concentrations of cytokines, growth factors, and nutrients in the portal vein, which promotes survival, proliferation, and differentiation of transplanted hPMSCs. In contrast, hPMSCs transplanted through peripheral paths induce fever and adverse immune reactions. Also, a significant portion of hPMSCs may be lost when circulating through the lung [61]. Therefore, only a small portion of transplanted hPMSCs entered the liver after circulation and immune damage, impeding the restoration of liver functions.

To determine whether transplanted MSC-derived cells colonized the pig liver, *alu *expression was detected by PCR. The *alu *repeat sequence is present only in humans and other primates. It accounts for 10% of the nucleotides in the human genome. The *alu *sequence is around 300-bp and is the most abundant sequence which appears at 3-4-kb intervals with moderate repetition. Because the *alu *family exhibits species-specificity, it can be used as a biomarker of human cells [62,63]. In our study, we amplified the *alu *sequence to determine whether hPMSCs were successfully transplanted into the pig liver. A specific 224-bp sequence was detected at a number of time points, demonstrating the presence of transplanted human MSCs in recipient pig livers.

## Conclusions

Taken together, our results suggest that MSCs from the human placenta could be used as an excellent source of transplanted cells for the repair of liver damage. Portal vein transplantation under B-ultrasonography guidance is safe and effective which could restore liver functions in pigs with liver failure to a substantial extent; however, the underlying mechanism remains to be further clarified.

## Abbreviations

AFP: alpha-fetoprotein; ALB: albumin; ALF: acute liver failure; ALP: alkaline phosphatise; ALT: alanine aminotransferase; APC: allophycocyanin; AST: aspartate aminotransferase; BSA: bovine serum albumin; CCl_4_: carbon tetrachloride; CHE: cholin esterase; CK18: cytokeratin 18; CYP450: cytochrome P450; DMEM: Dulbecco's modified eagle medium; EDTA: ethylene diamine tetraacetic acid; EGF: epidermal growth factor; ELISA: enzyme-linked immunosorbent assay; ELISpot: enzyme-linked immunospot; FGF: fibroblast growth factor; FITC: fluorescein isothiocyanate; GalN: D-galactosamine; HE: hematoxylin and eosin; HGF: hepatocyte growth factor; H_2_O_2_: hydrogen peroxide; hPMSCs: human placental mesenchymal stem cells; IFN: interferon; IMDM: Iscove's modified Dulbecco's medium; ITS: insulin-transferrin-selenium; LDL: low density lipoprotein; DiI-LDL: 1,19-dioctadecyl-3,3,39,39-tetramethylllindocarbocyane-low-density lipoprotein; LIF: leukemia inhibitory factor; LPL: lipoprotein lipase; MTT: 3-(4,5-dimethylthiazol-2-yl)-2,5-diphenyltetrazolium bromide; MNCs: mononuclear cells; MSCs: mesenchymal stem cells; NH_4_Cl: ammonium chloride; OD: optical density; OPN: osteopontin; PAS: periodic acid-schiff; PBS: phosphate buffered saline; PBMC: peripheral blood mononuclear cell; PCR: polymerase chain reactions; PE: phycoerythrin; TBA: total bile acid; TBIL: total bilirubin.

## Competing interests

The authors declare that they have no competing interests.

## Authors' contributions

All authors participated in interpretation of the findings. HC and LL were responsible for the conception and design of the study. JL and PZ generated the animal models. JY, JY, QP, and YW carried out cell biology and molecular biology experiments. YL and JL performed pathology tests. XP was responsible for the primary human hepatocytes culture and function analyses. HC, JY, and YW performed the statistical analysis and drafted the manuscript. All authors read and approved the final version of the paper. All authors confirm that the content has not been published elsewhere and does not duplicate their published work.

## Pre-publication history

The pre-publication history for this paper can be accessed here:

http://www.biomedcentral.com/1741-7015/10/56/prepub
